# Supplementary figures and images for: Small tropical islands with dense human population: differences in water quality of near-shore waters are associated with distinct bacterial communities
Source: PeerJ. 2018 May 7;6:e4555. doi: 10.7717/peerj.4555 (PMC5944435; doi:10.7717/peerj.4555)

**(A) FL**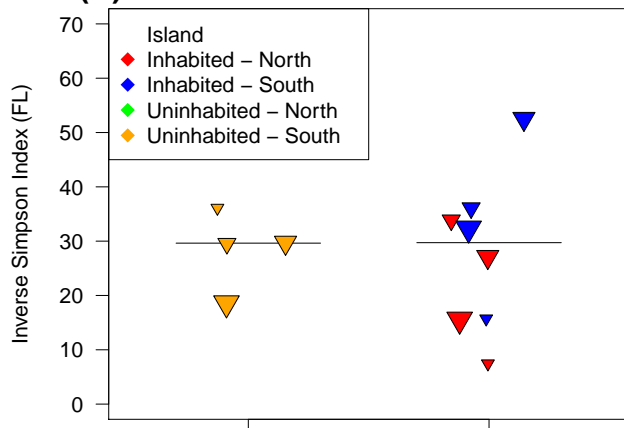**(B) FL**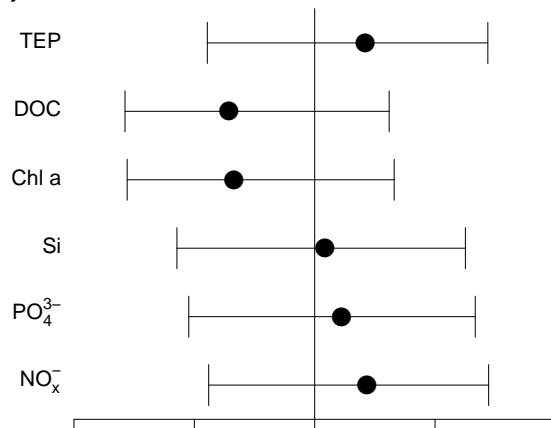**(C) PA**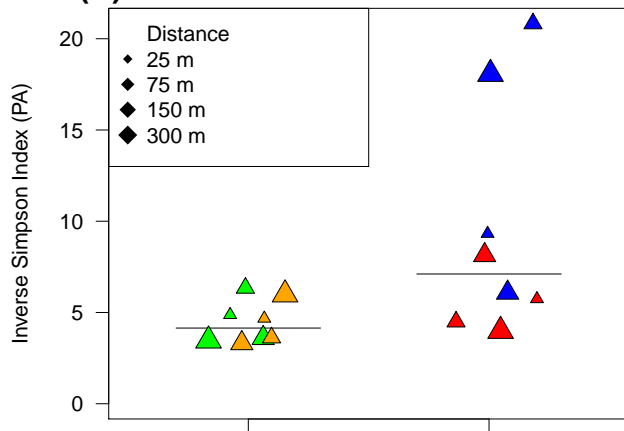**(D) PA**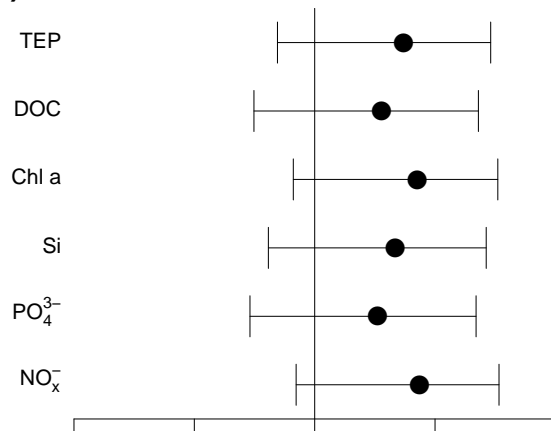**(E) SED**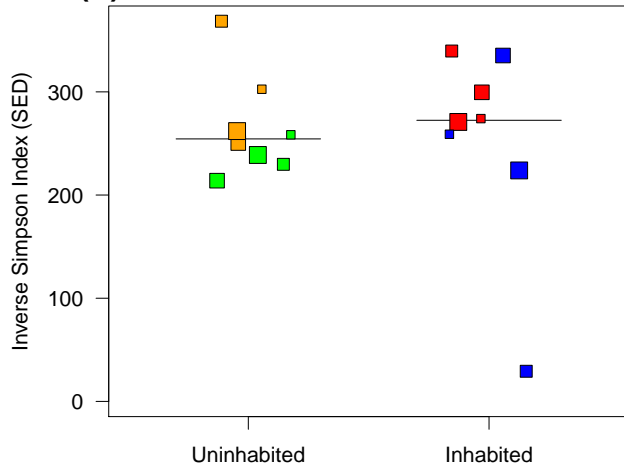**(F) SED**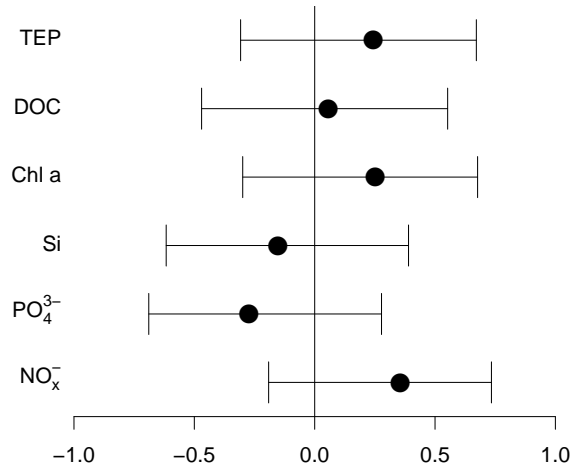

Supplement: Supplemental Information 2 — (A+B) Bacterial diversity of the free-living fraction of the water column (>0.2 μm; FL). (C+D) Bacterial diversity of the particle-attached fraction of the water column (>3 μm; PA). (E+F) Bacterial diversity in the reef sediment (SED). (A+C+E) Individual diversity estimates and their median (horizontal line) of the bacterial communities at each island. (B+D+F) Correlation coefficients based on Spearman-rank correlations with error bars depicting 95% confidence intervals. Inverse Simpson Indices were calculated based on repeatedly randomly rarefying the data set to the minimum library size (964 sequences). NOx−, nitrite/nitrate; PO43−, phosphate; Si, silicate; Chl a, Chlorophyll a; DOC, dissolved organic carbon; TEP, transparent exopolymer particles. [file peerj-06-4555-s002.pdf]

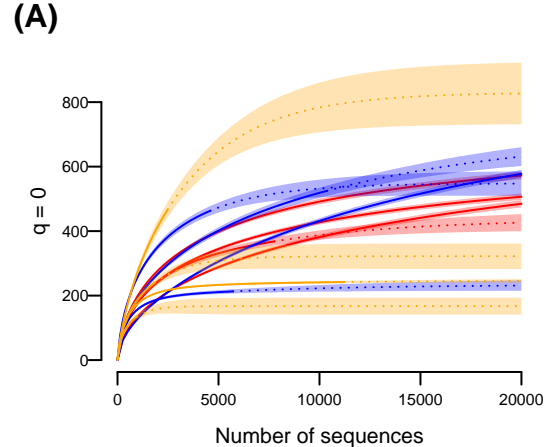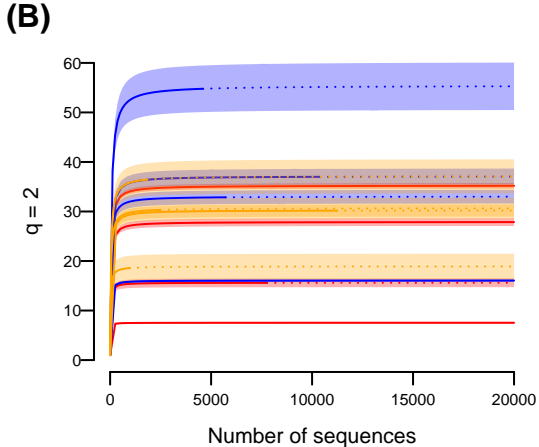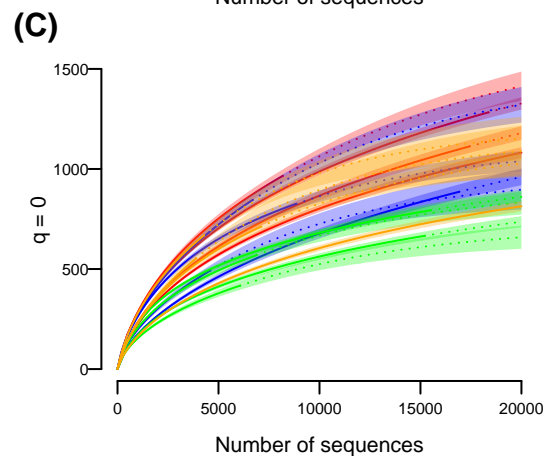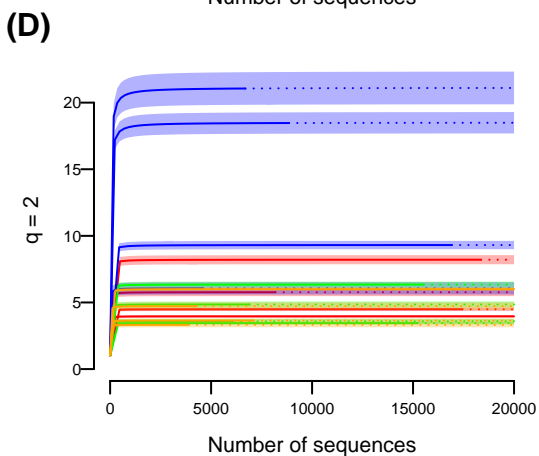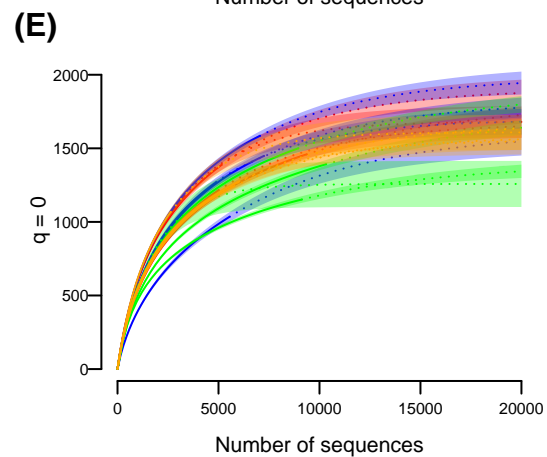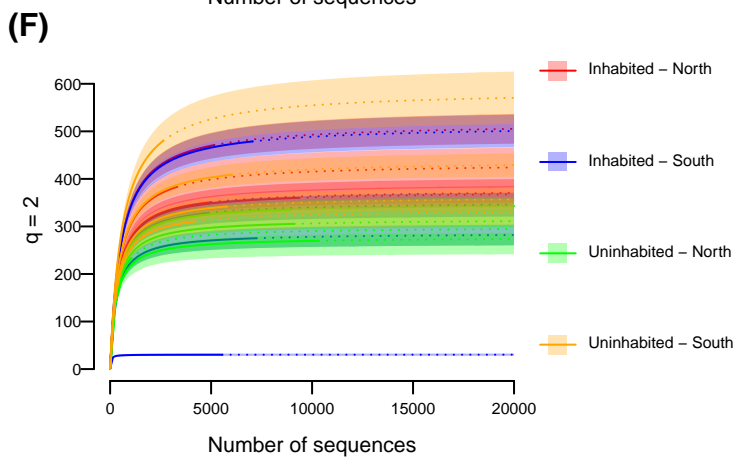

Supplement: Supplemental Information 3 — Rarefaction analysis of alpha diversity indices of bacterial communities at the inhabited and uninhabited island for sequencing depths of 0 to 20,000 sequences using the R packageiNEXT. (A+B) Bacterial diversity of the free-living fraction of the water column (>0.2 μm; FL). (C+D) Bacterial diversity of the particle-attached fraction of the water column (>3 μm; PA). (E+F) Bacterial diversity in the reef sediment (SED). (A+C+E) Number of OTUs (Hill number q = 0). (B+D+F) Inverse Simpson Index (Hill number q = 2). Solid lines: interpolated diversity indices, dashed lines: extrapolated diversity indices, shaded area: confidence interval of diversity estimates. [file peerj-06-4555-s003.pdf]

**(A)**

Bray-Curtis dissimilarity

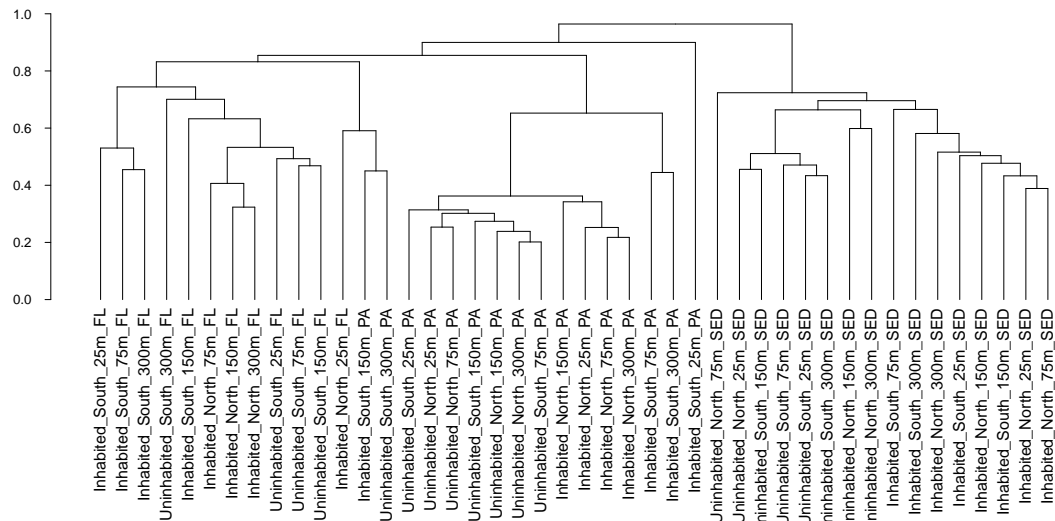**(B)**

Relative sequence abundance [%]

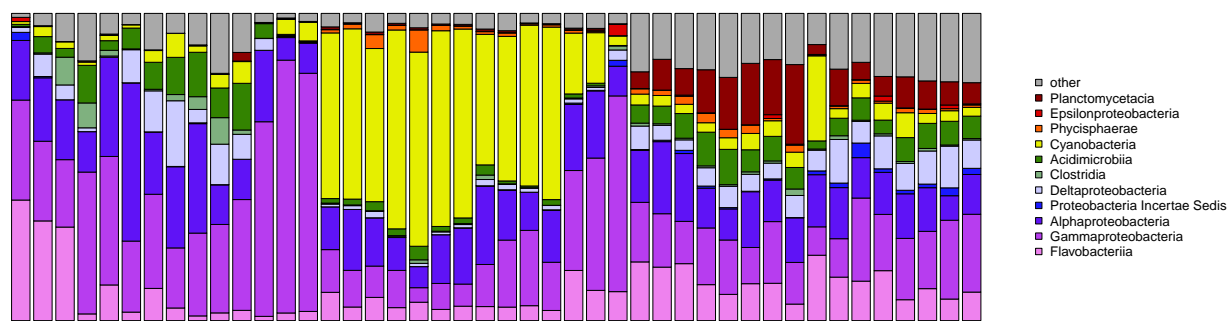

Supplement: Supplemental Information 4 — (A) Cluster diagram based on Bray-Curtis dissimilarity coefficients constructed using average linkage. (B) Class-level taxonomic composition of the bacterial communities. FL, Free-living bacterial communities of the water column (>0.2 μm); PA, Particle-attached bacterial communities of the water column (>3 μm); SED, Bacterial communities in reef sediments. [file peerj-06-4555-s004.pdf]

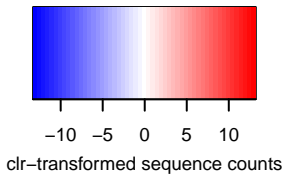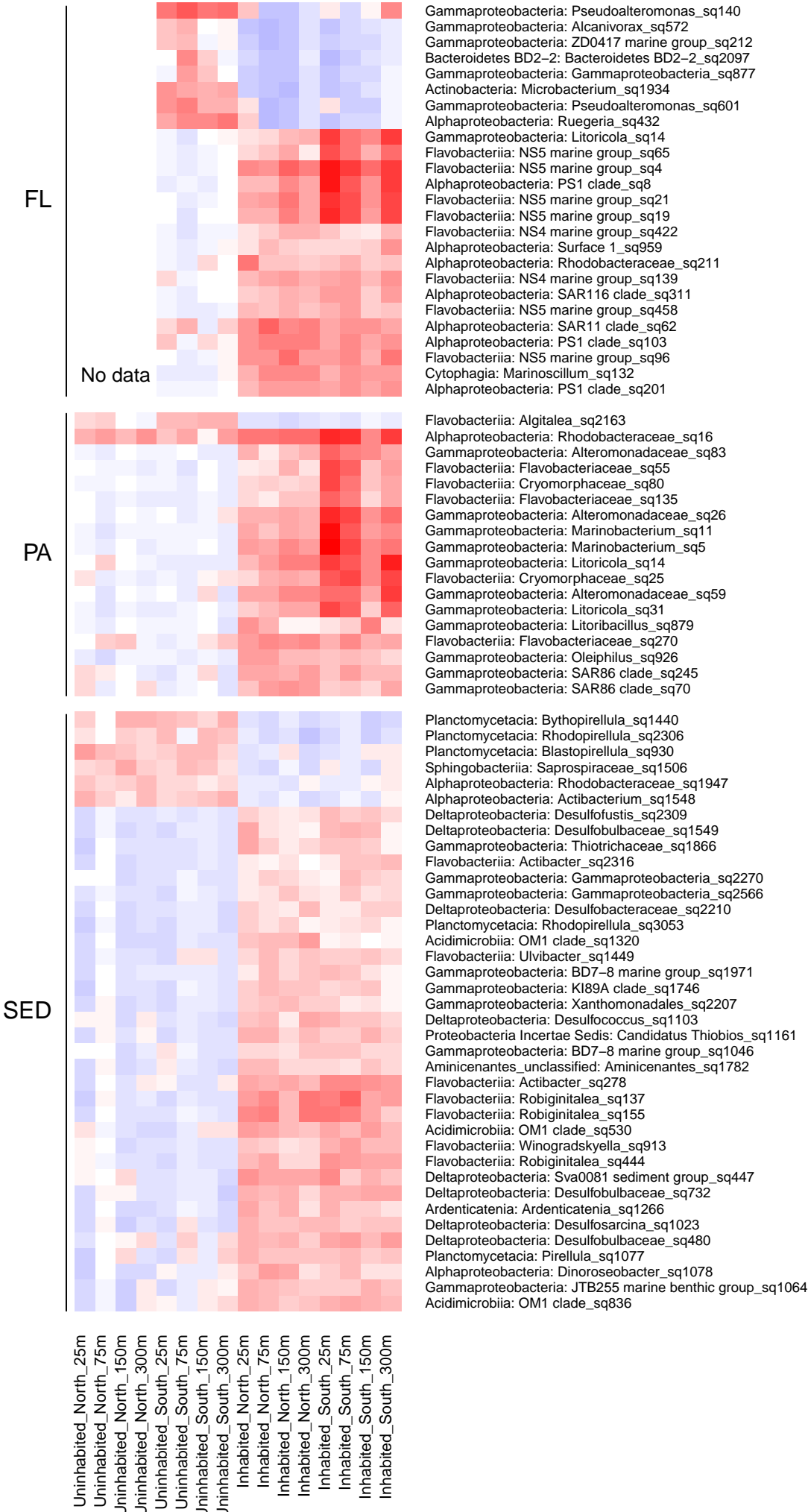

Supplement: Supplemental Information 5 — Red colors indicate an enrichment of an OTU compared to the average sequence contribution of all OTUs in a sample, whereas blue colors indicate depletion. For each OTU, its class-level affiliation, last classified taxonomic rank, and sequence number are provided. FL, Free-living bacterial communities of the water column (>0.2 μm); PA, Particle-attached bacterial communities of the water column (>3 μm); SED, Bacterial communities in reef sediments. [file peerj-06-4555-s005.pdf]

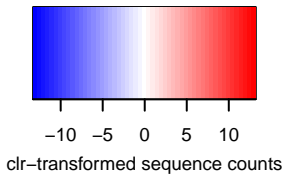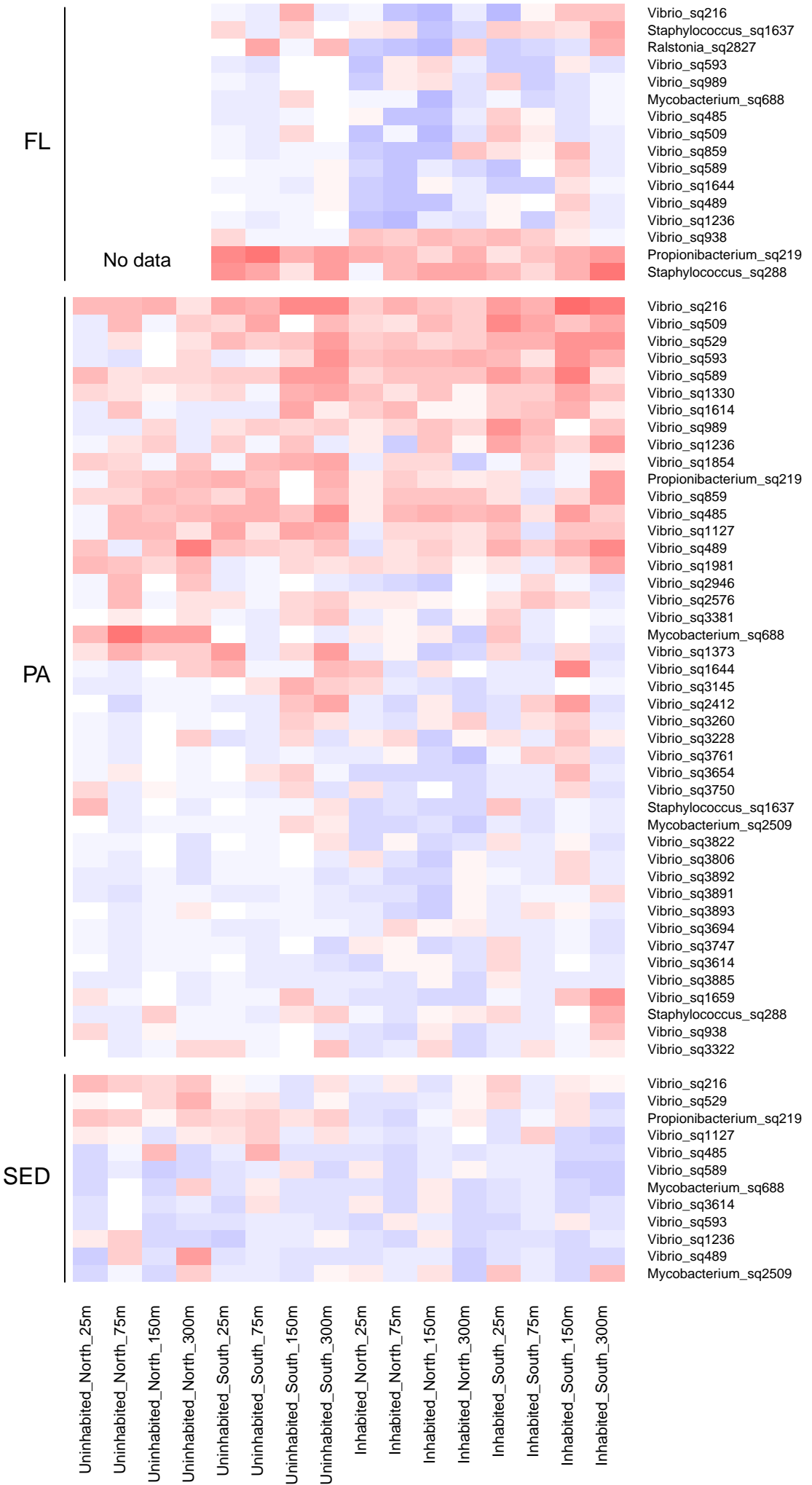

Supplement: Supplemental Information 6 — Red colors indicate an enrichment of an OTU compared to the average sequence contribution of all OTUs in a sample, whereas blue colors indicate depletion. For each OTU, its genus-level affiliation and sequence number are provided. FL, Free-living bacterial communities of the water column (>0.2 μm); PA, Particle-attached bacterial communities of the water column (>3 μm); SED, Bacterial communities in reef sediments. [file peerj-06-4555-s006.pdf]
