# Supplementary material for: Small tropical islands with dense human population: differences in water quality of near-shore waters are associated with distinct bacterial communities
Source: PeerJ. 2018 May 7;6:e4555. doi: 10.7717/peerj.4555 (PMC5944435; doi:10.7717/peerj.4555)
Supplement: Supplemental Information 7 — NOx−, nitrite/nitrate; PO43−, phosphate; Si, silicate; Chl a, Chlorophyll a; DOC, dissolved organic carbon; TEP, transparent exopolymer particles; N, number of replicates; SD, standard deviation; SEM, standard error of the mean. [file peerj-06-4555-s007.docx]

Supplementary table S1: Summary of the water quality parameters at the inhabited (BL: Barrang Lompo) and the uninhabited island (KK: Kodinggareng Keke). NO_x_^-^: nitrite/nitrate, PO_4_^3-^: phosphate, Si: silicate, Chl a: Chlorophyll a, DOC: dissolved organic carbon, TEP: transparent exopolymer particles, N: number of replicates, SD: standard deviation, SEM: standard error of the mean.

|  | Island | Transect | Distance [m] | N | Min | Max | Mean | SD | SEM |
| --- | --- | --- | --- | --- | --- | --- | --- | --- | --- |
| NO_x_^-^ [µmol L^-1^] | | |  |  |  |  |  |  |  |
|  | BL | North | 25 | 3 | 0.153 | 0.182 | 0.163 | 0.016 | 0.009 |
|  | BL | North | 75 | 3 | 0.226 | 0.275 | 0.243 | 0.027 | 0.016 |
|  | BL | North | 150 | 3 | 0.090 | 0.157 | 0.117 | 0.035 | 0.020 |
|  | BL | North | 300 | 3 | 0.071 | 0.097 | 0.083 | 0.013 | 0.008 |
|  | BL | South | 25 | 3 | 0.585 | 0.603 | 0.592 | 0.010 | 0.006 |
|  | BL | South | 75 | 3 | 0.160 | 0.177 | 0.170 | 0.009 | 0.005 |
|  | BL | South | 150 | 3 | 0.134 | 0.257 | 0.183 | 0.065 | 0.038 |
|  | BL | South | 300 | 3 | 0.132 | 0.165 | 0.149 | 0.017 | 0.010 |
|  | KK | North | 25 | 3 | 0.161 | 0.161 | 0.161 | 0.000 | 0.000 |
|  | KK | North | 75 | 3 | 0.161 | 0.161 | 0.161 | 0.000 | 0.000 |
|  | KK | North | 150 | 3 | 0.047 | 0.058 | 0.052 | 0.006 | 0.003 |
|  | KK | North | 300 | 3 | 0.043 | 0.106 | 0.066 | 0.035 | 0.020 |
|  | KK | South | 25 | 3 | 0.160 | 0.161 | 0.161 | 0.000 | 0.000 |
|  | KK | South | 75 | 3 | 0.161 | 0.161 | 0.161 | 0.000 | 0.000 |
|  | KK | South | 150 | 3 | 0.160 | 0.160 | 0.160 | 0.000 | 0.000 |
|  | KK | South | 300 | 3 | 0.161 | 0.161 | 0.161 | 0.000 | 0.000 |
| PO_4_^3-^ [µmol L^-1^] | | |  |  |  |  |  |  |  |
|  | BL | North | 25 | 3 | 0.133 | 0.135 | 0.134 | 0.001 | 0.001 |
|  | BL | North | 75 | 3 | 0.136 | 0.148 | 0.141 | 0.006 | 0.004 |
|  | BL | North | 150 | 3 | 0.131 | 0.137 | 0.133 | 0.003 | 0.002 |
|  | BL | North | 300 | 3 | 0.128 | 0.133 | 0.131 | 0.003 | 0.002 |
|  | BL | South | 25 | 3 | 0.178 | 0.185 | 0.181 | 0.004 | 0.002 |
|  | BL | South | 75 | 3 | 0.137 | 0.155 | 0.145 | 0.009 | 0.005 |
|  | BL | South | 150 | 3 | 0.137 | 0.139 | 0.137 | 0.001 | 0.001 |
|  | BL | South | 300 | 3 | 0.147 | 0.154 | 0.150 | 0.004 | 0.002 |
|  | KK | North | 25 | 3 | 0.083 | 0.087 | 0.084 | 0.002 | 0.001 |
|  | KK | North | 75 | 3 | 0.082 | 0.087 | 0.085 | 0.002 | 0.001 |
|  | KK | North | 150 | 3 | 0.137 | 0.140 | 0.138 | 0.002 | 0.001 |
|  | KK | North | 300 | 3 | 0.144 | 0.150 | 0.146 | 0.003 | 0.002 |
|  | KK | South | 25 | 3 | 0.087 | 0.110 | 0.096 | 0.012 | 0.007 |
|  | KK | South | 75 | 3 | 0.085 | 0.089 | 0.087 | 0.002 | 0.001 |
|  | KK | South | 150 | 3 | 0.096 | 0.097 | 0.097 | 0.000 | 0.000 |
|  | KK | South | 300 | 3 | 0.094 | 0.098 | 0.096 | 0.002 | 0.001 |
| Si [µmol L^-1^] | | |  |  |  |  |  |  |  |
|  | BL | North | 25 | 3 | 3.796 | 4.262 | 3.968 | 0.255 | 0.147 |
|  | BL | North | 75 | 3 | 4.067 | 4.387 | 4.195 | 0.169 | 0.098 |
|  | BL | North | 150 | 3 | 3.786 | 4.169 | 3.937 | 0.204 | 0.118 |
|  | BL | North | 300 | 3 | 3.649 | 4.014 | 3.801 | 0.190 | 0.110 |
|  | BL | South | 25 | 3 | 5.286 | 12.696 | 7.855 | 4.196 | 2.422 |
|  | BL | South | 75 | 3 | 3.846 | 4.929 | 4.209 | 0.623 | 0.360 |
|  | BL | South | 150 | 3 | 3.582 | 3.766 | 3.655 | 0.098 | 0.056 |
|  | BL | South | 300 | 3 | 4.803 | 5.033 | 4.924 | 0.115 | 0.067 |
|  | KK | North | 25 | 3 | 3.583 | 3.862 | 3.716 | 0.140 | 0.081 |
|  | KK | North | 75 | 3 | 3.482 | 3.920 | 3.640 | 0.243 | 0.140 |
|  | KK | North | 150 | 3 | 3.230 | 5.866 | 4.128 | 1.506 | 0.870 |
|  | KK | North | 300 | 3 | 3.487 | 3.869 | 3.736 | 0.216 | 0.125 |
|  | KK | South | 25 | 3 | 3.780 | 12.513 | 6.905 | 4.868 | 2.810 |
|  | KK | South | 75 | 3 | 3.583 | 3.780 | 3.653 | 0.110 | 0.064 |
|  | KK | South | 150 | 3 | 3.558 | 3.824 | 3.672 | 0.137 | 0.079 |
|  | KK | South | 300 | 3 | 3.551 | 3.894 | 3.721 | 0.171 | 0.099 |

Supplementary table S1 continued.

|  | Island | Transect | Distance [m] | N | Min | Max | Mean | SD | SEM |
| --- | --- | --- | --- | --- | --- | --- | --- | --- | --- |
| Chl a [µg L^-1^] | | |  |  |  |  |  |  |  |
|  | BL | North | 25 | 3 | 0.780 | 0.920 | 0.837 | 0.074 | 0.043 |
|  | BL | North | 75 | 3 | 0.750 | 0.830 | 0.787 | 0.040 | 0.023 |
|  | BL | North | 150 | 3 | 0.660 | 0.730 | 0.687 | 0.038 | 0.022 |
|  | BL | North | 300 | 3 | 0.490 | 0.570 | 0.520 | 0.044 | 0.025 |
|  | BL | South | 25 | 3 | 0.460 | 0.490 | 0.480 | 0.017 | 0.010 |
|  | BL | South | 75 | 3 | 0.430 | 0.490 | 0.463 | 0.031 | 0.018 |
|  | BL | South | 150 | 3 | 0.400 | 0.470 | 0.423 | 0.040 | 0.023 |
|  | BL | South | 300 | 3 | 0.440 | 0.490 | 0.463 | 0.025 | 0.015 |
|  | KK | North | 25 | 3 | 0.020 | 0.040 | 0.028 | 0.011 | 0.006 |
|  | KK | North | 75 | 3 | 0.020 | 0.030 | 0.023 | 0.006 | 0.003 |
|  | KK | North | 150 | 3 | 0.010 | 0.020 | 0.016 | 0.005 | 0.003 |
|  | KK | North | 300 | 3 | 0.010 | 0.020 | 0.014 | 0.005 | 0.003 |
|  | KK | South | 25 | 3 | 0.060 | 0.070 | 0.064 | 0.005 | 0.003 |
|  | KK | South | 75 | 3 | 0.000 | 0.000 | 0.000 | 0.000 | 0.000 |
|  | KK | South | 150 | 3 | 0.020 | 0.050 | 0.032 | 0.016 | 0.009 |
|  | KK | South | 300 | 3 | 0.000 | 0.050 | 0.023 | 0.025 | 0.015 |
| DOC [µmol L^-1^] | | |  |  |  |  |  |  |  |
|  | BL | North | 25 | 3 | 73.418 | 96.029 | 85.314 | 11.352 | 6.554 |
|  | BL | North | 75 | 3 | 66.715 | 87.038 | 73.777 | 11.492 | 6.635 |
|  | BL | North | 150 | 3 | 63.047 | 66.067 | 64.345 | 1.554 | 0.897 |
|  | BL | North | 300 | 3 | 74.232 | 87.891 | 81.595 | 6.892 | 3.979 |
|  | BL | South | 25 | 3 | 88.636 | 105.952 | 95.755 | 9.059 | 5.230 |
|  | BL | South | 75 | 3 | 72.935 | 86.014 | 77.617 | 7.288 | 4.208 |
|  | BL | South | 150 | 3 | 75.036 | 79.173 | 76.442 | 2.365 | 1.365 |
|  | BL | South | 300 | 3 | 67.297 | 77.495 | 72.537 | 5.105 | 2.947 |
|  | KK | North | 25 | 3 | 67.297 | 77.495 | 72.537 | 5.105 | 2.947 |
|  | KK | North | 75 | 3 | 65.983 | 70.942 | 68.382 | 2.483 | 1.434 |
|  | KK | North | 150 | 3 | 65.317 | 74.656 | 69.050 | 4.943 | 2.854 |
|  | KK | North | 300 | 3 | 72.504 | 78.678 | 74.860 | 3.337 | 1.926 |
|  | KK | South | 25 | 3 | 66.521 | 71.804 | 70.006 | 3.018 | 1.743 |
|  | KK | South | 75 | 3 | 67.144 | 70.667 | 69.426 | 1.978 | 1.142 |
|  | KK | South | 150 | 3 | 66.590 | 68.517 | 67.401 | 0.999 | 0.577 |
|  | KK | South | 300 | 3 | 72.153 | 78.000 | 75.218 | 2.934 | 1.694 |
| TEP [µg X_eq_ L^-1^] | | |  |  |  |  |  |  |  |
|  | BL | North | 25 | 3 | 99.163 | 152.752 | 128.018 | 27.031 | 15.607 |
|  | BL | North | 75 | 3 | 89.544 | 134.202 | 113.820 | 22.582 | 13.038 |
|  | BL | North | 150 | 3 | 68.246 | 122.522 | 96.186 | 27.174 | 15.689 |
|  | BL | North | 300 | 3 | 101.911 | 125.270 | 113.591 | 11.680 | 6.743 |
|  | BL | South | 25 | 3 | 149.317 | 231.761 | 184.356 | 42.591 | 24.590 |
|  | BL | South | 75 | 3 | 136.263 | 149.317 | 140.614 | 7.537 | 4.351 |
|  | BL | South | 150 | 3 | 132.141 | 148.630 | 140.614 | 8.254 | 4.765 |
|  | BL | South | 300 | 3 | 92.979 | 120.461 | 103.514 | 14.821 | 8.557 |
|  | KK | North | 25 | 3 | 56.108 | 66.414 | 60.918 | 5.187 | 2.995 |
|  | KK | North | 75 | 3 | 75.345 | 90.460 | 84.277 | 7.923 | 4.575 |
|  | KK | North | 150 | 3 | 45.116 | 95.957 | 74.429 | 26.300 | 15.184 |
|  | KK | North | 300 | 3 | 56.108 | 93.895 | 75.345 | 18.903 | 10.914 |
|  | KK | South | 25 | 3 | 88.399 | 135.118 | 114.507 | 23.839 | 13.764 |
|  | KK | South | 75 | 3 | 75.345 | 110.384 | 95.041 | 17.920 | 10.346 |
|  | KK | South | 150 | 3 | 97.331 | 119.316 | 108.094 | 11.000 | 6.351 |
|  | KK | South | 300 | 3 | 61.605 | 73.971 | 67.788 | 6.183 | 3.570 |
