# Supplementary material for: Small tropical islands with dense human population: differences in water quality of near-shore waters are associated with distinct bacterial communities
Source: PeerJ. 2018 May 7;6:e4555. doi: 10.7717/peerj.4555 (PMC5944435; doi:10.7717/peerj.4555)
Supplement: Supplemental Information 8 — NOx−, nitrite/nitrate; PO43−, phosphate; Si, silicate; Chl a, Chlorophyll a; DOC, dissolved organic carbon; TEP, transparent exopolymer particles. [file peerj-06-4555-s008.docx]

Supplementary table S2: List of eigenvectors of the individual water quality parameters contributing to the principal components (PC) of Fig. 3. NO_x_^-^: nitrite/nitrate, PO_4_^3-^: phosphate, Si: silicate, Chl a: Chlorophyll a, DOC: dissolved organic carbon, TEP: transparent exopolymer particles.

|  | PC1 | PC2 | PC3 | PC4 | PC5 | PC6 |
| --- | --- | --- | --- | --- | --- | --- |
| NO_x_^-^ | -0.44 | 0.37 | -0.32 | 0.28 | -0.21 | -0.67 |
| PO_4_^3-^ | -0.44 | -0.31 | 0.18 | -0.56 | 0.48 | -0.36 |
| Si | -0.28 | 0.68 | 0.47 | -0.36 | -0.23 | 0.25 |
| Chl a | -0.37 | -0.54 | 0.41 | 0.20 | -0.61 | 0.02 |
| DOC | -0.42 | -0.10 | -0.68 | -0.30 | -0.19 | 0.48 |
| TEP | -0.47 | 0.06 | 0.13 | 0.59 | 0.52 | 0.37 |
