# Supplementary material for: Small tropical islands with dense human population: differences in water quality of near-shore waters are associated with distinct bacterial communities
Source: PeerJ. 2018 May 7;6:e4555. doi: 10.7717/peerj.4555 (PMC5944435; doi:10.7717/peerj.4555)
Supplement: Supplemental Information 9 — OTU number (nOTU) and Inverse Simpson diversity index (invS) were calculated based on the complete data set as well as repeatedly randomly rarefying the data set to the minimum library size (964 sequences). [file peerj-06-4555-s009.docx]

Supplementary table S3: Number of generated (raw) and quality-checked (final) sequences produced from bacterial communities of the free-living and particle-attached fraction of the water column and reef sediment at the inhabited (BL: Barrang Lompo) and the uninhabited island (KK: Kodinggareng Keke). OTU number (nOTU) and Inverse Simpson diversity index (invS) were calculated based on the complete data set as well as repeatedly randomly rarefying the data set to the minimum library size (964 sequences).

| Habitat | Island | Transect | Distance [m] | nSEQ (raw) | nSEQ (final) | nOTU | nOTU (rarefied) | invS | invS (rarefied) |
| --- | --- | --- | --- | --- | --- | --- | --- | --- | --- |
| Free-living | |  |  |  |  |  |  |  |  |
|  | BL | North | 25 | 92695 | 43803 | 604 | 133.05 | 7.53 | 7.45 |
|  | BL | North | 75 | 63918 | 36086 | 564 | 172.79 | 35.21 | 33.85 |
|  | BL | North | 150 | 55383 | 27100 | 602 | 196.34 | 27.87 | 27.11 |
|  | BL | North | 300 | 16571 | 7764 | 368 | 176.58 | 15.59 | 15.57 |
|  | BL | South | 25 | 94828 | 49329 | 758 | 124.74 | 16.06 | 15.72 |
|  | BL | South | 75 | 20263 | 10376 | 525 | 188.62 | 37.00 | 36.09 |
|  | BL | South | 150 | 9988 | 4593 | 463 | 252.38 | 54.78 | 52.53 |
|  | BL | South | 300 | 11507 | 5715 | 213 | 156.13 | 32.86 | 32.33 |
|  | KK | South | 25 | 4471 | 1833 | 240 | 186.26 | 36.44 | 36.11 |
|  | KK | South | 75 | 23724 | 11228 | 242 | 174.02 | 30.17 | 29.54 |
|  | KK | South | 150 | 6978 | 2487 | 467 | 267.71 | 30.31 | 29.73 |
|  | KK | South | 300 | 2645 | 964 | 135 | 135.00 | 18.57 | 18.57 |
| Particle-attached | | |  |  |  |  |  |  |  |
|  | BL | North | 25 | 25533 | 8179 | 965 | 276.33 | 5.77 | 5.74 |
|  | BL | North | 75 | 49246 | 17436 | 1113 | 228.06 | 4.49 | 4.50 |
|  | BL | North | 150 | 58403 | 18366 | 1283 | 272.37 | 8.21 | 8.14 |
|  | BL | North | 300 | 75441 | 27418 | 1223 | 216.78 | 3.96 | 3.99 |
|  | BL | South | 25 | 47875 | 16916 | 886 | 172.31 | 9.31 | 9.32 |
|  | BL | South | 75 | 16384 | 6691 | 845 | 265.01 | 21.06 | 20.83 |
|  | BL | South | 150 | 18644 | 4597 | 493 | 194.24 | 6.06 | 6.08 |
|  | BL | South | 300 | 20839 | 8853 | 826 | 254.33 | 18.47 | 18.07 |
|  | KK | North | 25 | 19575 | 6898 | 570 | 200.79 | 4.85 | 4.87 |
|  | KK | North | 75 | 56618 | 15525 | 794 | 222.22 | 6.34 | 6.34 |
|  | KK | North | 150 | 18898 | 6070 | 417 | 150.98 | 3.59 | 3.59 |
|  | KK | North | 300 | 41136 | 15214 | 666 | 167.68 | 3.45 | 3.46 |
|  | KK | South | 25 | 12891 | 4280 | 668 | 272.43 | 4.72 | 4.67 |
|  | KK | South | 75 | 20249 | 7110 | 712 | 224.25 | 3.64 | 3.62 |
|  | KK | South | 150 | 14500 | 3887 | 553 | 224.81 | 3.32 | 3.31 |
|  | KK | South | 300 | 65080 | 24000 | 873 | 159.05 | 6.00 | 5.98 |
| Sediment | |  |  |  |  |  |  |  |  |
|  | BL | North | 25 | 20091 | 5006 | 1215 | 534.54 | 351.14 | 274.08 |
|  | BL | North | 75 | 20463 | 5149 | 1387 | 585.52 | 471.03 | 339.58 |
|  | BL | North | 150 | 11752 | 3312 | 1178 | 588.93 | 383.17 | 299.59 |
|  | BL | North | 300 | 30276 | 9362 | 1459 | 519.14 | 360.10 | 270.83 |
|  | BL | South | 25 | 21438 | 4880 | 1269 | 546.40 | 328.34 | 258.93 |
|  | BL | South | 75 | 19953 | 5550 | 1035 | 382.37 | 30.18 | 29.23 |
|  | BL | South | 150 | 27250 | 7048 | 1585 | 594.94 | 478.76 | 335.24 |
|  | BL | South | 300 | 24319 | 7244 | 1448 | 536.26 | 275.14 | 223.85 |
|  | KK | North | 25 | 27851 | 8945 | 1500 | 537.94 | 335.49 | 258.40 |
|  | KK | North | 75 | 4993 | 1047 | 574 | 542.68 | 232.69 | 229.77 |
|  | KK | North | 150 | 29536 | 10321 | 1392 | 483.84 | 269.82 | 213.94 |
|  | KK | North | 300 | 30225 | 9123 | 1152 | 461.51 | 305.43 | 238.83 |
|  | KK | South | 25 | 20696 | 6023 | 1275 | 540.31 | 408.70 | 302.56 |
|  | KK | South | 75 | 9963 | 2633 | 1067 | 600.36 | 480.27 | 368.48 |
|  | KK | South | 150 | 13328 | 4224 | 1118 | 522.61 | 310.73 | 250.42 |
|  | KK | South | 300 | 17811 | 5785 | 1243 | 530.92 | 341.89 | 261.99 |
