# Supplementary material for: Small tropical islands with dense human population: differences in water quality of near-shore waters are associated with distinct bacterial communities
Source: PeerJ. 2018 May 7;6:e4555. doi: 10.7717/peerj.4555 (PMC5944435; doi:10.7717/peerj.4555)
Supplement: Supplemental Information 10 — Performance of the random forest analysis to predict the inhabitation status of the islands based on their microbial communities. Each model was run with 10001 trees. [file peerj-06-4555-s010.docx]

Supplementary table S4: Performance of the random forest analysis to predict the inhabitation status of the islands based on their microbial communities. Each model was run with 10001 trees.

|  | **Free-living** | **Particle-attached** | **Sediment** |
| --- | --- | --- | --- |
| Number of variables tried at each split | 28 | 43 | 48 |
| Model out-of-bag error | 0.083 | 0.063 | 0.063 |
| Random out-of-bag error | 0.417 | 0.625 | 0.625 |
| p-value | < 0.001 | < 0.001 | < 0.001 |
| Accuracy^a^ | 0.917 | 0.938 | 0.938 |
| Kappa^a^ | 0.824 | 0.875 | 0.875 |
| Lower cut-off mean decrease in accuracy^b^ | 0.0020 | 0.0030 | 0.0016 |
| Lower cut-off mean decrease in GINI^b^ | 0.040 | 0.075 | 0.040 |

^a^ determined by leave-one-out cross-validation

^b^ criterion for variable importance
